# Nature vs. nurture: FOXP3, genetics, and tissue environment shape Treg function

**DOI:** 10.3389/fimmu.2022.911151

**Published:** 2022-08-12

**Authors:** Arielle Raugh, Denise Allard, Maria Bettini

**Affiliations:** ^1^ Department of Pathology, Microbiology and Immunology, University of Utah, Salt Lake City, UT, United States; ^2^ Translational Biology and Molecular Medicine Graduate Program, Baylor College of Medicine, Houston, TX, United States

**Keywords:** Treg - regulatory T cell, T cell, autoimmunity, type 1 diabetes, genetic, FOXP3

## Abstract

The importance of regulatory T cells (Tregs) in preventing autoimmunity has been well established; however, the precise alterations in Treg function in autoimmune individuals and how underlying genetic associations impact the development and function of Tregs is still not well understood. Polygenetic susceptibly is a key driving factor in the development of autoimmunity, and many of the pathways implicated in genetic association studies point to a potential alteration or defect in regulatory T cell function. In this review transcriptomic control of Treg development and function is highlighted with a focus on how these pathways are altered during autoimmunity. In combination, observations from autoimmune mouse models and human patients now provide insights into epigenetic control of Treg function and stability. How tissue microenvironment influences Treg function, lineage stability, and functional plasticity is also explored. In conclusion, the current efficacy and future direction of Treg-based therapies for Type 1 Diabetes and other autoimmune diseases is discussed. In total, this review examines Treg function with focuses on genetic, epigenetic, and environmental mechanisms and how Treg functions are altered within the context of autoimmunity.

## Introduction

At the crossroads of autoimmunity and health are regulatory T cells (Tregs) - a crucial immune cell involved in tolerance towards self and suppression of auto-antigen specific T cells. Tregs were first identified as a subpopulation of CD4 T cells that expressed the high affinity IL-2 receptor chain CD25 (1). However, it took several more years to identify the lineage specific transcription factor, Forkhead Box Protein 3 (FOXP3), that is a core regulator of suppressive Treg function (2–4), and acts as both a positive and negative regulator of gene expression (2–4). For example, FOXP3 directly upregulates CD25 expression, but suppresses IL-2 production (5). With the knowledge of how to identify Tregs and a basic understanding of their function, the field was propelled towards key findings regarding their developmental source, suppressive mechanisms, and therapeutic potential (6–8).

While the transcription factor FOXP3 was initially considered the “master regulator” of CD4 Treg development and function (7, 8) we now understand that a more complex system is at work. Rather than a single element, the Treg suppressive program is regulated by a combination of transcription factors, genetic and epigenetic elements, as well as tissue-microenvironment cues. Due to the complexity that underlies the Treg suppressive phenotype, it has become apparent that loss of Treg lineage commitment can occur through either loss of FOXP3 or through a number of alternative genetic and/or transcriptional dysregulations. However, the precise alterations that occur in autoimmune individuals that affect Treg-mediated tolerance, and how underlying genetic variations impact the development and function of Tregs during autoimmunity are only partially elucidated. Polygenetic susceptibly is a key driving factor of many autoimmune diseases. However, while genome wide association studies (GWAS) alone were suggestive, they were not sufficient to formally link Treg dysfunction to disease. Integration of GWAS studies with functional and other omics-data now implicate alterations or defects in regulatory T cell function in autoimmune pathogenesis (9–11).

In this review we consider the function and regulation of FOXP3 both during homeostasis and autoimmunity, as well as how FOXP3 and mutations in key Treg genes influence Treg function and stability. In addition, we examine epigenetic modifications that regulate FOXP3 activity and how inflammation in the surrounding tissue environment impacts Tregs. Finally, we feature how Treg based therapies for autoimmunity have changed since their inception as well as factors that need to be improved in order to make these therapies efficacious as treatments for autoimmunity.

## Mutations in the FOXP3 gene

**Figure 1:**
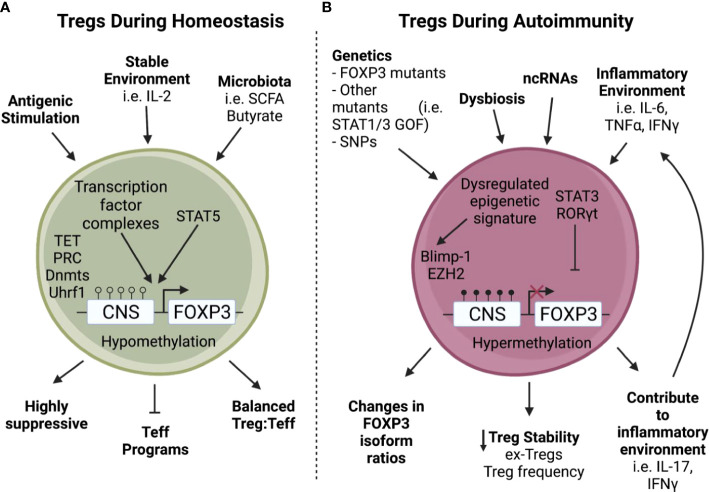
Tregs encounter increased stressors during autoimmunity. **A)** During homeostasis, Tregs are stimulated through TCR activation and proliferate using IL-2 from the surrounding environment (1, 5), allowing downstream transcription factor complexes to bind to a hypomethylated Foxp3 and enact critical Treg functions such as suppressive capabilities and repression of Teff programming (15–18). **B)** In contrast, Tregs found during autoimmunity often have intrinsic defects in addition to environmental stressors (2, 12–14, 19–23). For example, non-coding RNAs can be dysregulated during autoimmunity (24–26) leading to a dysregulated epigenetic signature with increased methylation of the Foxp3 Treg Specific Demethylated Region (TSDR) which can cause a loss of Foxp3 (27). In addition, a tissue environment rich in inflammatory cytokines can convert Tregs into Th17-like cells leading to the creation of ex-Tregs and decreased Treg stability (32–34). Furthermore, these stressors encountered during autoimmunity can also lead to perturbations in FOXP3 isoform ratios (35, 36) and expression of inflammatory cytokines (32, 33) which in turn leads to decreased Treg stability.

Immune dysregulation, polyendocrinopathy, enteropathy X-linked (IPEX) syndrome is a rare disorder that often results from mutations within the *FOXP3* gene (2, 12, 13). However, in a cohort of 173 patients with IPEX syndrome symptoms, only 50.9% had direct mutations in *FOXP3*, underscoring the fragility of Treg function and its sensitivity to modulation of alternative pathways (14). Of the 85 patients that had no discernable *FOXP3* mutation, 25% had mutations in key Treg genes such as *LRBA, STAT1, STAT3, CTLA4, IL2RA, STAT5B*, and *DOCK8* which are responsible for various aspects of Treg differentiation and function. This suggests that although FOXP3 is critical for Treg mediated tolerance, other factors also participate in maintaining a functional Treg population (**Figure 1A**). For example, mice lacking the inhibitory molecule CTLA4 develop severe lymphoproliferative disease reminiscent of *Foxp3* mutant mice (37, 38). Furthermore, another study of 15 IPEX patients bearing *FOXP3* mutations revealed that Treg signature genes were still expressed, although with variable expression levels, indicating that Tregs can still maintain partial lineage characteristics after loss of FOXP3 expression (28). A transcriptomic disease signature was observed across both Tregs and conventional CD4 cells and was likely induced by global immune dysregulation. To put it differently, transcriptomic changes occur as a result of both cell-intrinsic and cell-extrinsic mechanisms, where Tregs first have dysregulated core genes involved in Treg stability and suppressive function *(i.e. Il2ra, Tnfrsf4, Tnfrsf9, Tnfrsf18, Capg, Ikzf2, and Ctla4)*, which in turn alter the tissue environment, ultimately leading to enhanced broad transcriptomic changes affecting all T cells (28). In the absence of cell-extrinsic inflammatory signals in heterozygous mothers of IPEX patients, patient *FOXP3* mutations impacted only a narrow set of genes directly under FOXP3 control. In combination, these observations point to limited direct impacts of *FOXP3* mutations and an increased role for activation of inflammatory feedback loops leading to cumulative dysregulation of both regulatory and effector T cells. This further underscores the importance of Tregs’ ability to integrate information from their environment and alter their subsequent functions.

FOXP3 has four structural domains that are used to interact with diverse binding partners to exert transcriptional regulation. The examination of *FOXP3* mutations in IPEX patients and in mouse models has provided important insights into the function of the specific domains within FOXP3. Mutations in *FOXP3* identified in IPEX patients have been localized to all four structural domains of the transcription factor, although to some level they are concentrated in the DNA-binding FKH domain (29). For example, identification of a patient with a mutation within the dimerization motif in the FKH domain of *FOXP3* showed that FOXP3’s domain swap interface is crucial for restricting Th2 immune responses in Tregs. When the domain swap interface is mutated, FOXP3 interacts with Th2 specific loci inducing expression of Th2 signature cytokines that are normally repressed in Tregs (29).

While most mutations within *FOXP3* result in systemic immune dysregulation and global autoimmune manifestations, partial disruption of interactions between FOXP3 and its binding partners can have understated effects on FOXP3-driven gene activity. Foxp3-GFP reporter mice that express GFP fused to Foxp3 at its N-terminus provided a system to observe how subtle changes can have disease-specific impacts (30, 31). The Foxp3-GFP reporter mouse shows no abnormal Treg function on the C57BL/6 genetic background; however, when backcrossed to the NOD autoimmune-susceptible strain it resulted in rapidly accelerated autoimmune diabetes development (30, 31). Foxp3-GFP showed reduced interaction with several binding partners involved in *Foxp3* gene regulation, suggesting Foxp3 instability and loss of Treg function under increased inflammatory stress (30). Interestingly, the GFP-modified Foxp3 was protective in a model of arthritis due to disruption in HIF1α binding and increasing Foxp3 interactions with Interferon Regulatory Factor 4 (IRF4) leading to improved Treg control of Th2 and Th17 responses (31). Perturbations in Treg function were observed in autoimmune prone, but not autoimmune resistant mice suggesting that the genetic or inflammatory environment has a direct influence on the ultimate functionality of Tregs. The loss of Treg stability under inflammatory conditions has been a concern in situations of chronic autoimmunity and has been directly observed in mouse models of autoimmune diabetes, multiple sclerosis, and rheumatoid arthritis (32–34). Loss of Foxp3 expression in these situations resulted in the formation of ‘ex-Tregs’ that acquired an effector pro-inflammatory phenotype (**Figure 1B**). However, such ex-Tregs have not been directly observed in human autoimmune conditions, and if they exist are more likely to be localized directly in inflammatory tissues.

## Polygenetic susceptibilities and Treg function

Autoimmune manifestations that result from direct mutations of the *FOXP3* gene and related Treg functional genes, such as *CTLA4*, can be traced to loss of Treg numbers and/or function (38–40). However, it has been more challenging to infer the target immune cell population in polygenetic autoimmune susceptibilities. HLA alleles associated with autoimmunity contribute the largest risk for development of autoimmunity, including type 1 diabetes (9). For some HLA alleles, such as DQ8 and DQ2, loss of self-tolerance is thought to be prompted by the structure of the peptide binding grooves, which lead to increased selection or peripheral activation of autoimmune T cells (41). Many other T1D associated SNPs are located in close proximity to immune genes, such as *CTLA4*, and components of the IL-2 and TCR signaling pathways among others. The cytokine IL-2 binds to CD25 (*IL2RA*) and signals through STAT5 to regulate FOXP3 expression in Tregs (**Figure 1A**) (42). Complete *IL2RA* deficiency can lead to severe autoimmunity with IPEX like symptoms (43), and *IL2RA* variants have been associated with reduced Treg numbers, suboptimal Treg function, and an increased risk for development of T1D (44). Since HLA alleles, *CTLA4*, and *IL2RA* among others are implicated in both T effector (Teff) and Treg function, the ultimate impact on either population is difficult to determine. Nevertheless, several T1D related SNPs have been connected to Treg function (45–47) and the Treg to T effector cell ratio (48). Additionally, evidence suggests that Tregs from T1D patients may not be as suppressive and may have a more inflammatory phenotype (49). Therefore, there is a growing consensus that Treg function is altered in T1D, and Treg dysregulation might be in part due to genetics.

In many other autoimmune and inflammatory disorders it is not so clear whether there is an underlying defect in regulatory T cells. Many polymorphic variants are shared between several autoimmune diseases, including PTPN22 (TCR signaling), TKY2 (cytokine signaling), and TNFAIP3 (TNF signaling) among others (50, 51). These variants point to genes besides FOXP3 that could influence T cell and Treg function during autoimmunity. For example, in the context of rheumatoid arthritis there is still an ongoing debate regarding Treg dysfunction. There are a number of conflicting observations on whether Treg frequency decreases or remains stable (52–55), whether there are changes in Treg suppressive capability, or the relative expression of Treg associated regulatory molecules, such as CTLA-4 (52, 56, 57). The markers used to define Tregs as well as disease severity should be carefully considered in these studies, and could potentially explain some of the discrepancies in observations. Nevertheless, the lack of clear loss in Treg number or function in RA supports the idea that Treg dysfunction is disease specific.

The majority of disease-associated genetic variants defined by GWAS studies are found in non-coding areas of the genome, which presents a challenge in determining the ultimate relationship between SNPs, gene expression, and downstream effects on cellular function. Importantly, many disease associated SNPs are mapped to regulated chromatin regions and enhancers, i.e. epigenetically regulated transcription factor binding sites (58–60). Several mechanisms for non-coding regions’ impact on immune genes have been described. These range from direct disruption of transcription factor binding at SNPs located within enhancer regions (61) to distal effects mediated by genomic misfolding and interconnection of enhancers in 3D chromatin organization (62). Recent studies have coupled epigenetic profile analyses of isolated T cell populations to determine the effects of particular SNPs on chromatin accessibility in the context of T cell populations. Interestingly, the chromatin accessibility at these loci is preferentially associated with naive and activated Tregs, rather than conventional T cells (10, 63, 64). These observations imply that genetic susceptibility disproportionally effects Treg function compared to effector T cells in the context of autoimmunity. Based on cumulative genetic studies we can infer that genetic polymorphisms have connections to FOPX3+ Treg function and predisposition to autoimmunity (9, 44–48). Therefore, it is critical to examine the transcriptional regulation of the Treg lineage and the factors that impinge on Treg stability.

## Genetic regulation of the FOXP3 locus

Genetic control and regulation of *FOXP3* plays a major role in Treg development and function during both homeostasis and disease. While several FOXP3 isoforms have been identified in humans, there are two distinct isoforms that are necessary for optimal Treg function; the full length FOXP3 isoform and the alternatively spliced FOXP3 isoform which lacks exon 2 (FOXP3Δ2) (65). The full length FOXP3 isoform has recently been identified as a critical component of regulating FOXP3 activity and maintaining Treg stability (66). FOXP3Δ2 on the other hand, has been shown to be upregulated during Treg activation, and is linked to transcription of the transmembrane protein, Glycoprotein A Repetitions Predominant (GARP), which tethers TGFβ to the cell membrane and potentiates cell-contact dependent TGFβ function (67, 68). While both isoforms are necessary for optimal Treg function (65), regulation of FOXP3 isoform ratios appears to alter the disease course in some autoimmune diseases (**Figure 1B**) (35, 36).

Regulation of the *FOXP3* locus is multifaceted and involves several key enhancer regions that recruit a number of regulators that control Treg development and stabilize the Treg lineage (**Figure 1A**). The *FOXP3* locus has four enhancer regions known as conserved non-coding sequences (CNS; CNS0, CNS1, CNS2, and CNS3) that work in tandem to drive *FOXP3* transcription and downstream gene expression necessary for Treg stability (15–17, 69, 70). These enhancer regions are embedded throughout upstream-promoter and intronic regions of *FOXP3* (71, 72) and alter *FOXP3* transcription and activity by controlling methylation status, chromatin accessibility, and act as docking sites for unique sets of binding partner complexes (15–17, 73, 74). For example, the transcription factor SATB1 binds CNS0 (18) which along with the transcription factor HIVEP2 co-regulates pathways involved in Treg immunosuppression (75). SATB1 is an important transcription factor in regulating T cell differentiation (76); however, it is repressed by FOXP3 in Tregs to balance Treg proliferation and function. Loss of SATB1 increases Treg frequency but diminishes Treg suppressive function (77, 78). In Tregs, *SATB1* is epigenetically regulated through histone trimethylation and acetylation changes, as well as by microRNAs such as mir-155, mir-21a, mir-7, mir-34a, and mir-18a (79). During development, IL-2 signaling directs the pioneer factor SATB1 to bind nucleosome dense regions in Tregs leading to chromatin remodeling and accessibility of critical Treg signature genes (77). This is aided by the transcription factor Foxp1 which enhances IL-2 signaling and Foxp3 expression (78), making IL-2 signaling a critical step in differentiating Tregs from CD25+Foxp3- Treg precursors in the thymus (15).

CNS1 is primarily associated with peripheral induction of Tregs and is bound by several transcription factors including AP-1, NFAT, Foxo1, Hhex, Batf3, and importantly Smad3 induced by TGFβ signaling. Batf3 represses FOXP3 expression and downregulates the differentiation of naïve CD4 T cells into Tregs (80). In addition, Hhex (Hematopoietically expressed homeobox) is a transcription factor that binds to CNS1/CNS2 and represses FOXP3 expression; particularly under inflammatory conditions (81). CNS2 is a critical response element during thymic Treg development, and is bound by Ets-1, CREB, Stat5, NFAT, c-Rel, Runx, Foxp3, and AP-1. Importantly, CNS2 contains the Regulatory T cell Specific Demethylated Region (TSDR) (82), which maintains FOXP3 expression in Tregs and allows FOXP3 to positively regulate its own transcription even in the absence of TCR signaling (18). Lastly, CNS3 is another region important for the development of thymic Tregs and can bind Foxo and c-Rel (17, 83–85). These transcription factor binding complexes can alter FOXP3 activity, downstream targets of FOXP3, and additional pathways involved in Treg function (75). In addition, CNS regions CNS0 and CNS3, were recently determined to be sites that help initiate Treg development when bound by transcription factor complexes that allow chromatin remodeling and drive FOXP3 transcription (16). Beyond FOXP3 enhancer regions, transcription of Treg signature genes is also regulated by cooperation of Foxp3 and one of the five transcription factors Eos, IRF4, GATA-1, Lef1, and Satb1. These cofactors, referred to as the “quintet”, enhance Foxp3 activity by ‘locking in’ and stabilizing Foxp3 to its binding sites (18).

Furthermore, demethylation status of the *FOXP3* TSDR was determined to be key for maintaining FOXP3 expression and stabilizing Treg identity. However, while demethylation of the TSDR is enough to stabilize FOXP3 expression in Tregs, it is not enough to confer suppressive function (86). This suggests that Treg suppressive function is not solely linked to FOXP3 expression, and that additional transcription factors are required. As an example, the transcription factor Helios is expressed in approximately 70% of Tregs and helps to maintain Treg stability by controlling certain aspects of Treg function, differentiation, and survival (87). However, mice lacking Helios are still able to convert naïve T cells into functional Tregs; indicating a level of redundancy in transcriptional regulation of Treg function (88).

## Heterogeneity within the Treg population

**Figure 2:**
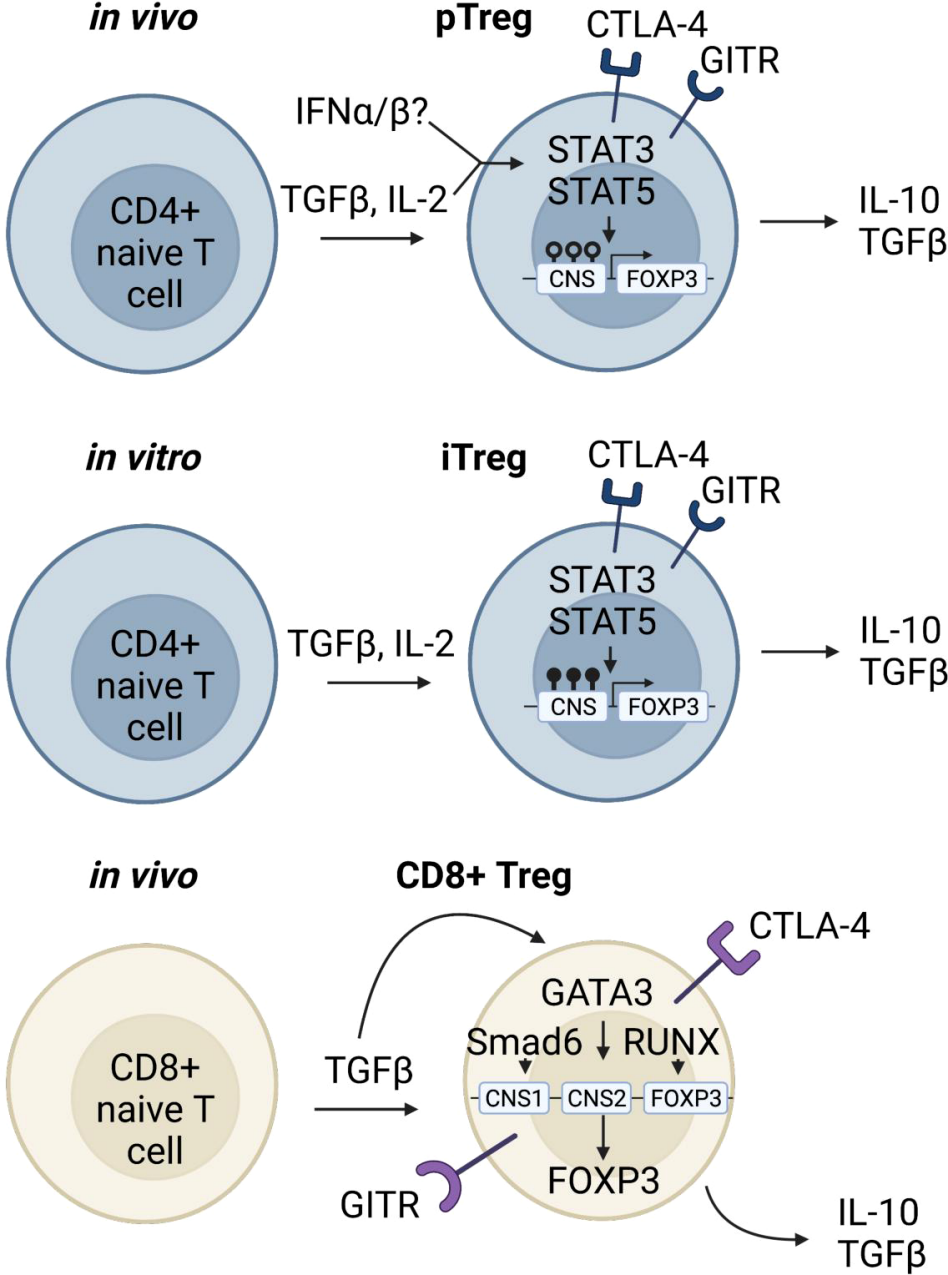
Induced regulatory T cells. In the periphery, T lymphocytes can encounter stimuli that turn on downstream signaling leading to genetic reprogramming and a regulatory phenotype (90, 91). pTregs which have the capacity to be immunosuppressive and traffic to inflamed tissue sites are differentiated from CD4 naïve T cells under inflammatory conditions (92–94). A rare and unique subpopulation of Tregs is the CD8+Foxp3+ Treg. In the periphery, when naïve CD8 Tconv cells encounter TGFβ, pSmad3 binds to CNS1 of *Foxp3*, and along with transcription factors Runx3 and Gata3 promote expression of Foxp3 (95–97). CD8+Foxp3+ Tregs express similar markers as CD4 Tregs and have immunosuppressive functions.

The FOXP3+ Treg population exhibits phenotypic and functional complexity driven by tissue and context specific transcription factors. Similar to conventional T cells (Tconv), the majority of lymphoid derived thymic Tregs maintain a non-activated phenotype, characterized by expression of CD62L, CCR7 and TCF1 (a transcription factor associated with stemness) (89). However, Tregs can also be derived from naïve CD4 T cells in the periphery through TGFβ signaling (90, 91) (**Figure 2**). TGFβ signal can be provided in the form of latent TGFβ on the cell surface of tTregs, which leads to induction of additional Foxp3+ T cells (pTregs), in a process that is described as “infectious tolerance” (**Figure 2**) (98). Upon differentiation from naïve T cells, *in vivo* induced pTregs repress CD4 effector T cell programming, stabilize expression of FOXP3, and maintain a fully demethylated TSDR, similar to tTregs (92–94). In addition, recent work suggests that type 1 interferons can stabilize expression of STAT3, STAT5, and FOXP3 in peripheral CD4 T cells allowing their differentiation into pTregs (**Figure 2**) (99). However, Type 1 interferons have been shown to have opposing effects on Tregs depending on the timing of exposure. In the short-term, Type 1 interferons lead to decreased Treg frequency and function; however, in the long run they can stabilize expression of FOXP3 and promote Treg expansion (100). Nevertheless, since no definitive markers of pTregs have been identified, the functional importance of pTregs during autoimmunity is still heavily debated (101–103).

The widely accepted approach to induce Treg differentiation *in vitro* relies on a combination of TCR ligation in the context of TGFβ and high concentrations of IL-2 (**Figure 2**) (104). While studies show that iTregs have suppressive function both *in vitro* and *in vivo*, their long-term stability is more controversial (93, 105). Stability is measured by quantification of methylation at the TSDR region, and TCR and IL-2 stimulation can promote demethylation of TSDR in iTregs, thus stabilizing the lineage (106, 107). However, iTregs that have a hypermethylated TSDR can still be functional (108, 109).

**Figure 3:**
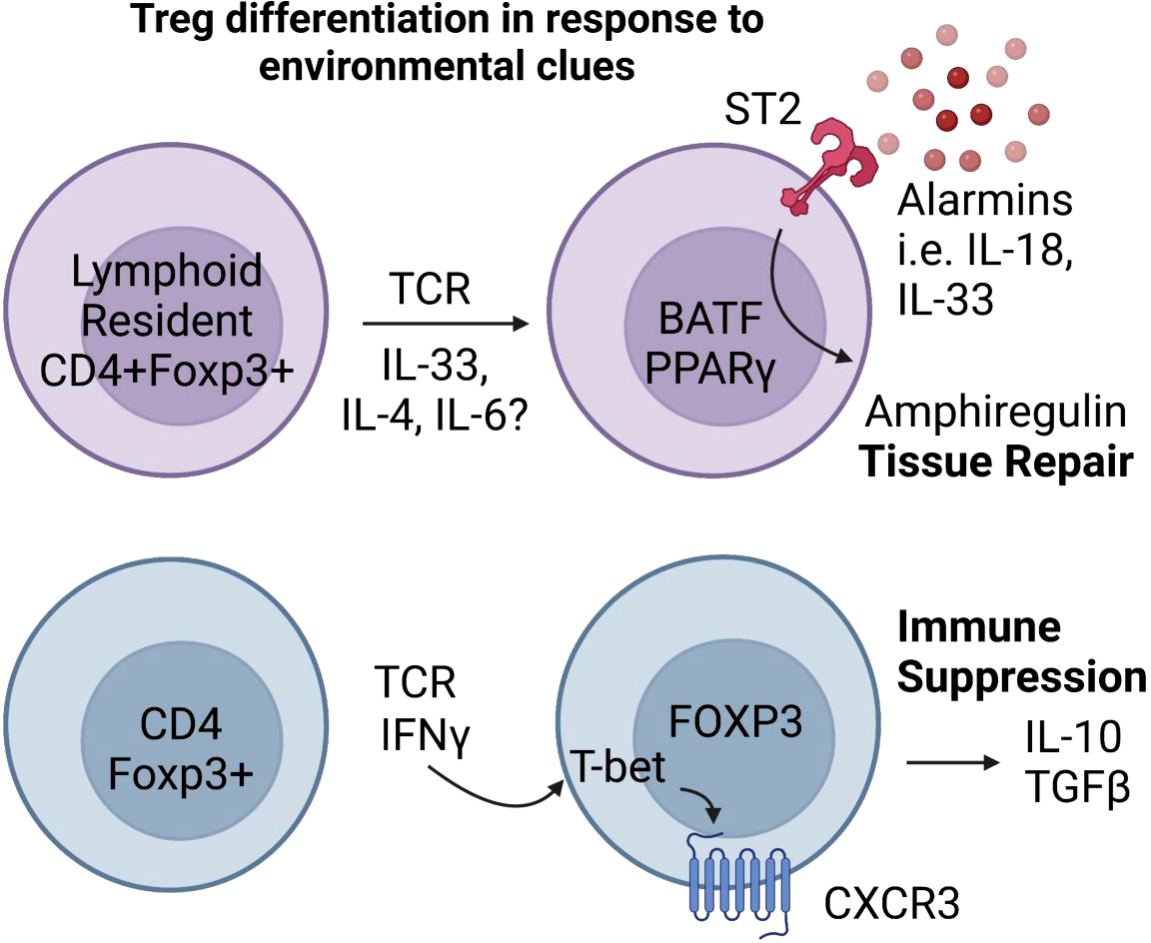
Inflammatory and tissue specific signals shape Treg responses. Tissue Tregs are poised to respond to inflammatory tissue environments. In the presence of alarmin cytokines, tissue Tregs expressing the transcriptional regulators Batf and PPARγ (89, 110–113) secrete the wound repair factor Amphiregulin (114–116). Inflammatory cytokines that normally drive T-helper lineage specific factors like T-bet can similarly induce T-helper transcription factor expression in Tregs. IFNγ and TCR stimulation induce T-bet expression in Foxp3+ Tregs, which provides them with increased ability to suppress Th1 effector T cells (117–119).

As Tregs migrate from lymphoid organs to peripheral tissues they accumulate a common tissue-resident signature and are further differentiated into unique phenotypes dependent on tissue-specific signals. These tissue-resident Tregs (tissue Tregs) have the potential to be derived from both tTregs and pTregs, with the change from a lymphoid-resident phenotype to a tissue-resident phenotype, a process that is mediated by a combination of transcriptional regulators (**Figure 3**) (111, 112). In the spleen and lymph nodes, the transcription factor BATF drives the stepwise progression of tissue Treg precursors into tissue Tregs by increasing chromatin accessibility of tissue specific Treg genes (113). Repression of BATF impairs tissue Treg function and contributes to induction of autoimmunity (120). In addition, tissue Tregs often exhibit specialized functions associated with upregulation of tissue specific transcription factors, such as PPARγ in visceral fat tissue and Eos in the skin (89, 110). Although, more recently PPARγ has been linked to skin and liver Tregs as well (121, 122). Interestingly, upregulation of IL-33R (ST2) and its downstream target cytokine, amphiregulin, is a trait shared among many Tregs that are transitioning towards tissue phenotype; indicative of an acquired ability to participate in tissue repair in response to inflammation or injury (**Figure 3**) (89, 123–125). The growth factor amphiregulin is expressed by tissue Tregs in response to alarmin cytokines released by injured tissue cells, including IL-33 (114–116). The ramifications of this discovery show that Tregs upregulate receptors necessary to sense the tissue microenvironment in order to rapidly respond to environmental changes.

In addition, CD4 T-helper lineage defining transcription factors can shape Treg responses during inflammation. A prime example of this is T-bet, which in addition to being the major Th1 lineage-defining transcription factor, provides Tregs with increased ability to suppress Th1 effectors (117). T-bet is upregulated in Tregs in response to IFNγ and TCR ligation and is directly responsible for the upregulation of chemokine receptor CXCR3, allowing Tregs to traffic to sites of inflammation (**Figure 3**) (118, 119).

Furthermore, CD8+FOXP3+ regulatory T cells constitute a smaller proportion of the Treg compartment but are still functional contributors to the regulatory arm of the immune system. They are transcriptionally similar to CD4+FOXP3+ T cells; and although they seem to be less potent than CD4+FOXP3+ Tregs they have been shown to be effective in models of GVHD and lupus (126, 127). In contrast to CD4+FOXP3+ T cells, CD8 Treg suppressor programs are controlled by the transcription factors RUNX3 and GATA3 (**Figure 2**). In naïve CD8 T cells, GATA3 binds to the CNS1 region of *FOXP3* to inhibit FOXP3 expression, however in CD8 Tregs, GATA3 binds to the CNS2 region to maintain FOXP3 expression (95). Furthermore, RUNX3 binds to the promoter region of *FOXP3* to initiate transcription, and under conditions with high levels of TGFβ, Smad3 is phosphorylated and binds to CNS1 inducing FOXP3 expression (**Figure 2**). nCD8+CD25+ Tregs are also somewhat functionally similar to CD4+FOXP3+ Tregs as they express suppressive markers such as GITR and CTLA4 (96), as well as cytokines such as IL-10 and TGFβ (**Figure 2**) (95, 97).

## Epigenetic regulation of Treg lineage

Epigenetic regulation of gene expression can have major consequences for cells. During development, thymocytes that are fated to become Tregs undergo a series of epigenetic modifications to CNS regions of *FOXP3* to activate transcription of *FOXP3* and downstream Treg signature genes. At the same time, T cell effector cellular differentiation programs are repressed (**Figure 1A**) (128, 129). Stepwise histone tail acetylation at the *FOXP3* promoter initiates chromatin remodeling and *FOXP3* transcription (130). *FOXP3* histone tail acetylation allows ten-eleven translocation (TET)-mediated DNA demethylation to occur in the CNS2 region of the *FOXP3* locus and maintains *FOXP3* transcription by increasing chromatin accessibility; thus, removing the need for further histone acetylation (130). Positive regulation of Treg lineage is also accomplished by repression of alternative T-helper lineage programs. Polycomb-repressive complexes (PRC) are multi-protein enzymes that transcriptionally silence genes through histone H2A ubiquitylation and H3K27 methylation (131). PRCs silence Th17 related genes and enhance the Wnt signaling pathway to favor Treg development and stability (132, 133).

Once chromatin remodeling and access to core Treg genes is achieved, additional epigenetic changes occur that maintain stable chromatin accessibility. For example, the protein ubiquitin like with PHD and ring finger domains 1 (Uhrf1) is an epigenetic regulator that recruits DNA methyltransferases (Dnmt) such as Dnmt1, Dnmt3a, and Dnmt3b to stabilize methylation patterns (134–137) during Treg development (**Figure 1A**), as well as following TCR engagement in the periphery (138). Similarly, ablation of Dnmt1 in Tregs severely impairs their function through global changes in methylation (139).

However, many of these normal epigenetic modifications fail to function and/or maintain Treg stability during autoimmunity, as Tregs derived from autoimmune patients often have epigenetic and transcriptomic changes. For example, effector Tregs derived from juvenile idiopathic arthritis patients present with consistent changes that include methylation changes in enhancer regions, as well as upregulation of functional and core Treg genes (140). The upstream regulatory elements that are dysregulated can be numerous due to the complexity of epigenetic mechanisms that control Treg lineage. For instance, in a model of multiple sclerosis, methylation of CNS2 normally repressed by Dnmt3a and controlled by Blimp1 is disrupted and leads to loss of Treg identity (141). There are indications that similar disruptions occur in human autoimmunity. The chromatin-modifying enzyme Ezh2 maintains Treg identity after activation, and its reduction is observed in RA patients (**Figure 1B**) (142, 143). Moreover, tissue antigens themselves can produce variable epigenetic responses in antigen-specific Tregs. For example, Tregs expanded *in vitro* using APCs expressing insulin B:9-23 peptide were found to have transcriptomic and epigenetic signatures representative of highly suppressive Tregs compared to Tregs expanded using whole insulin peptide (144). This provides evidence for the importance of T cell receptor signaling and antigen specificity in the development of optimally functional and stable Tregs.

Understanding the epigenetic changes that Tregs undergo during chronic inflammation is important for gaining new targeting strategies for autoimmune therapies. Tregs function differently during homeostasis and acute infection compared to chronic inflammatory conditions, implying context and inflammation specific Treg functional programs potentially regulated at the epigenetic level (19–21). In addition, Treg frequency and core signature gene expression mainly associated with DNA accessibility, transcription, translation, signal transduction, and cytokine receptors are prone to changing throughout the span of autoimmune disease pathology (145).

Microbiota have also been shown to influence Treg function and stability. Interestingly, some microbial-derived signals directly engage with Treg epigenetic elements (146). While still a new field of study, there is increasing evidence that short chain fatty acids (SCFA), such as butyrate, can be produced by commensal bacteria and positively regulate Treg differentiation (147). This appears to be CNS1 dependent, and is mediated by enhanced acetylation at the *FOXP3* locus (148, 149). However, it is still unclear whether SCFAs are the key signal for pTreg induction in the mesenteric lymph nodes (150). Importantly, gut dysbiosis is a feature of several autoimmune diseases such as IBD, SLE, RA, Graves’ Disease and T1D, and it might contribute to disbalance of immune homeostasis (**Figure 1B**) (151–156).Thus, it is relevant to ask if inflammation or other microenvironmental cues at tissue sites can play a direct role in changing Treg function through epigenetic and/or transcriptomic changes.

## Shifting the Treg/Teff equilibrium

A major question that remains regarding Tregs in autoimmunity is how they inevitably fail throughout the course of disease. One hypothesis is a decrease in the ratio of Tregs : Teffs, which can be seen in several autoimmune diseases (157–160). The shifts seen in this equilibrium could be the result of direct mutations in *FOXP3* such as in IPEX syndrome, other polymorphisms that affect Treg function or stability, or could occur due to the influence of the surrounding tissue environment, since normal cellular mechanisms of differentiation and function that work to maintain the Treg : Teff balance are often dysregulated during autoimmunity (**Figure 1**).

For example, a major pathway that diverts CD4 T cells away from Treg differentiation and towards a Th17 program is the IL-6/STAT3 pathway commonly associated with inflammation. Dysregulation of the IL-6/STAT3 pathway seen in patients with gain of function mutations in STAT3 is correlated with increased susceptibility to T1D; most likely related to the Treg : Th17 imbalance seen in these patients (22). Inflammatory environments high in IL-6 have been shown to increase the Th17 transcription factor RORγt in both tTregs and pTregs (23), and lead to the creation of ex-Tregs that are capable of secreting inflammatory cytokines (32, 33). These ex-Treg cells lose FOXP3 expression and convert into pathogenic Th17 cells capable of producing IFNγ and destabilizing Tregs in the surrounding environment (33, 161–163). Formation of ex-Tregs promotes a shift in the Treg : Teff ratio skewed towards destructive Teff cells. Treg-derived IFNγ can also act as a negative feedback regulator of Treg stability and lead to further loss of suppressive function, indicating an important role for the environment in continually shaping and sometimes destabilizing Treg responses (164). Additionally, antigen exposure and/or scarcity can impact the balance between Th17 and Treg differentiation (165). Recent evidence suggests that T cells can trogocytose MHCII molecules from APCs displaying specific antigens, and subsequently display the MHCII to other antigen-specific T cells. When differentiation is favored towards Tregs there is a high APC:T cell ratio, however, when the reverse occurs (high T cell:APC ratio) differentiation is skewed towards Th17 cells (166).

These inflammatory pathways implicated in Treg lineage destabilization can be effectively targeted for therapeutic purposes. Small molecule targeted inhibition of IL-6 or STAT3 promotes Treg development and leads to the establishment of homeostasis between Treg and Th17 cells in a model of multiple sclerosis (MS) (167, 168). MS patients often exhibit dysregulated cytokine levels - including an increase in IL-6 in their cerebral spinal fluid, which could be targeted with the goal of shifting the balance between anti- and pro-Treg micro-environment cues (169). However, blocking the IL-6R in early onset T1D patients with a mAb did not prevent or delay beta cell loss (170), illuminating the limitations of therapies that target a single inflammatory pathway.

## Non-coding RNAs during autoimmunity

Evidence shows dysregulated microRNA (miRNA) and long non-coding RNA (lncRNA) expression is also associated with many autoimmune diseases (24, 25). miRNAs are small non-coding RNAs that regulate proteins largely by binding to the 3’ UTR of mRNA and preventing translation, or by targeting the mRNA for degradation. Similarly, lncRNAs modulate chromatin architecture and mRNA stability (171). Both miRNA and lncRNA can impact Treg genetic regulation by altering expression of epigenetic regulators, directly targeting *FOXP3*, and by altering the signaling pathways that allow Tregs to respond to the surrounding microenvironment. Through these mechanisms, miRNAs influence Treg frequency and modify Treg functional capabilities.

Further, miRNAs can play an important role in regulating Treg epigenetics. For example, miR-142-3p, which is upregulated during T1D in humans and mice, can bind to lysine demethylase 6A (KDM6A) and demethylate H3K27me3 in Tregs leading to increased autophagy, decreased apoptosis, and increased Treg function (172). While two different Treg-specific miR-142 deficient mouse models showed impaired Treg function, whether or not Treg frequency is altered in these mice remains unclear since the two studies showed conflicting results (173, 174). Furthermore, miR-142-3p function in Tregs may operate through multiple pathways as miRNA142-3p also destabilizes Tregs by interacting with TET2 to alter Treg methylation in both humans and mice (27).

In addition to modifying Treg epigenetic signatures, non-coding RNAs can target *FOXP3* and other Treg signature genes. In humans, several miRNAs including, mi-R206, miR-133a, miR-133b, and miR-31 have been identified that directly target the 3’ UTR of *FOXP3* mRNA leading to FOXP3 translational downregulation (175, 176). miR-31 is among the better studied miRNAs that target *FOXP3* and has been implicated in numerous autoimmune diseases. In murine models of autoimmunity, mi-R31 is upregulated upon TCR stimulation, but is inhibited by TGFβ/NF-κB signaling (177). miR-31 functions by directly targeting *FOXP3*, and also acts indirectly by promoting HIF1α and downregulating Nrp1 and retinoic acid-inducible protein 3 (Gprc5a) (178). miR-31 also inhibits carcinoembryonic antigen related cell adhesion molecule 1 (CEACAM1)-S, which represses Treg development in a model of murine liver autoimmunity but promotes Treg development in peripheral blood mononuclear cells (PBMCs) isolated from systemic lupus erythematosus (SLE) patients (179). The ultimate effect of miR-31 on Treg development and frequency depends on the balance between its inhibitory and enhancer functions. However, the factors that determine this require further investigation.

In addition, miRNAs can also influence FOXP3 by targeting pathways that regulate its expression. miR-21, which is among the best studied miRNAs that regulate Tregs in this manner, is dysregulated in several autoimmune disorders in both humans and mice. miR-21 acts indirectly to positively regulate Foxp3 expression (180); however, in autoimmunity, reduced miR-21 expression is correlated with increased STAT3 and reduced STAT5 and Foxp3 expression (26, 181–183). miR-21 directly targets STAT3 resulting in its downregulation and subsequently reduces effector molecules IL-17 and IL-22 (182, 184). Maresin 1 (MaR1) and the EGF/c-Jun pathway have both been shown to induce miR-21, restore Treg : Teff ratios through FOXP3 induction, and reduce autoimmunity (183, 185).

While some studies show that transfection of naïve human CD4 T cells with miR-21 is sufficient to induce Treg development by increasing Foxp3, TGFβ, and IL-10, another study found that miR-21 promotes RORγt and suppresses Foxp3 and IL-10 (180, 186, 187). Indeed, Treg specific depletion of miR-21 in mice induced the expression of both IL-17 and IL-10 indicating that miR-21 may play a role in opposing pathways (184). In line with these opposing observations, increased miR-21 expression inhibited FOXP3+ Tregs in human gastric cancer (188) whereas it induced FOXP3 in human and mouse autoimmunity (182, 183). Interestingly, LPS stimulation of PBMCs from RA patients down-regulated miR-21; however, PBMCs from healthy controls responded to LPS in the opposite fashion by up-regulating miR-21 (175). The opposite regulation and effects of miR-21 in autoimmune patients compared to healthy controls and in cancer settings suggests that a complex network of factors determines whether miR-21 promotes or inhibits Treg stability and function.

Furthermore, lncRNAs can also modulate Treg epigenetics. For example, FOXP3 long intergenic noncoding RNA, *Flicr*, reduces chromatin accessibility to the CNS3/Accessible Region 5 in mature Tregs and represses FOXP3 expression in both humans and mice. Knockout of *Flicr* on the NOD mouse background results in stabilized Foxp3 expression with a reduction in diabetes incidence (25). Additionally, in both humans and mice the lncRNA lnc-Smad3 interacts with the histone deacetylase HDAC1 to silence SMAD3 transcription. Upon TGFβ stimulation SMAD3 inhibits lnc-Smad3, thus allowing for greater SMAD3 transcription (189).

lncRNAs are also integral in regulating key Treg transcription factors. For example, Homeobox D gene cluster antisense growth-associated long noncoding RNA (HAGLR) is another lncRNA involved in autoimmunity. In human Tregs, HAGLR suppresses RUNX3 expression resulting in reduced Treg frequency (190). Additionally, lncRNA DQ786243 induces FOXP3 expression in human Tregs and promotes Treg suppressive function (191).

Noncoding RNAs are also important participants in regulating and responding to environmental cues. In inflammatory environments rich in IL-6 and TNFα, NF-κb upregulates the expression of miR-34a in humans and mice (192), which attenuates FOXP3 expression and can result in a shift of the Treg : Teff ratio. miR-124, which is dysregulated in numerous autoimmune diseases (24, 26), inhibits IL-6/STAT3 signaling and promotes Treg development (193). Similarly, miR-146a normally targets *STAT5b* to enhance Treg function and differentiation, but loss of miR-146a during inflammatory conditions leads to reduced FOXP3 expression and reduced Treg frequency. IL-2 represses *Flicr* thus removing *Flicr’s* inhibition of FOXP3 expression, while TGFβ inhibits the Foxp3-repressive noncoding RNAs miR-31 and lnc-Smad3 (194). The anti-inflammatory molecules MaR1 and EGF promote miR-21 (183, 185). The field of noncoding RNAs and their role in Treg development and function is growing, but additional studies are still required to reveal the full extent they may have in autoimmunity.

## Treg-based therapies

With the central role for regulatory T cells in autoimmune diseases, it is unsurprising that investigation is underway as to how Tregs can be used therapeutically (195). One example is the use of Tregs as a treatment for T1D. The current standard of care for T1D patients is exogenous replacement of insulin. When managed well, the administration of synthetic insulin results in more stable blood glucose levels but does not entirely negate the risk of comorbidities (196). Thus, having an immunomodulatory therapy that prevents, attenuates, or reverses the course of pancreatic islet destruction is crucial.

## Altering Treg to Teff ratio using immunomodulation

Due to potential imbalance in the Treg : Teff homeostasis seen during T1D, much attention has been focused on changing the ratio either by depleting effector T cells or expanding the Treg population. One of the earliest immunomodulatory therapies attempted in T1D patients was the use of anti-CD3 antibodies (197–199). Even a single dose of anti-CD3 lessened T1D progression and allowed reduction or complete withdrawal from exogenous insulin replacement therapy in some patients (197). Following initial positive observations in early diagnosed patients, anti-CD3 mAb therapy was used in a clinical trial of relatives of T1D patients who had at least two diabetes related auto-antibodies and confirmed dysglycemia prior to the start of the trial (200). A subgroup of participants in the treatment arm of the trial displayed delayed onset of T1D compared to controls, showing that modulation of T cell function after loss of tolerance but prior to overt disease can influence disease outcomes. Anti-CD3 antibodies appear to function by altering the ratio of Tregs : Teffs, as Teffs are susceptible to depletion by anti-CD3, whereas Tregs are more resistant (201). Additionally, following anti-CD3 mAb therapy a temporary increase in PD1+FOXP3+ Tregs was seen that paralleled a rise in anergic/exhausted CD4 and CD8 Teff cells (202). While early versions of anti-CD3 mAbs resulted in significant side effects that limited their use, genetic engineering and proteolytical removal of Fc domains alleviated many of the side effects (203, 204). The recent successes obtained with the anti-CD3 mAb therapy in T1D allow us to conclude that (1) immunotherapeutic interventions can be successful in T1D, (2) timing of immunotherapy is important, but success can be achieved even after anti-beta cell responses are detected, and (3) shifting the balance between inflammatory and regulatory pathways might be sufficient to acquire long-term tolerance. Although anti-CD3 mAb therapy is highly promising, it is not effective for ~25% of T1D patients and its positive effects can be temporary, which necessitates further investigation of the mechanisms underlying persistence of autoimmune T cells and their resistance to anti-CD3 therapy in certain individuals (197, 198).

## Direct expansion of Tregs

**Figure 4:**
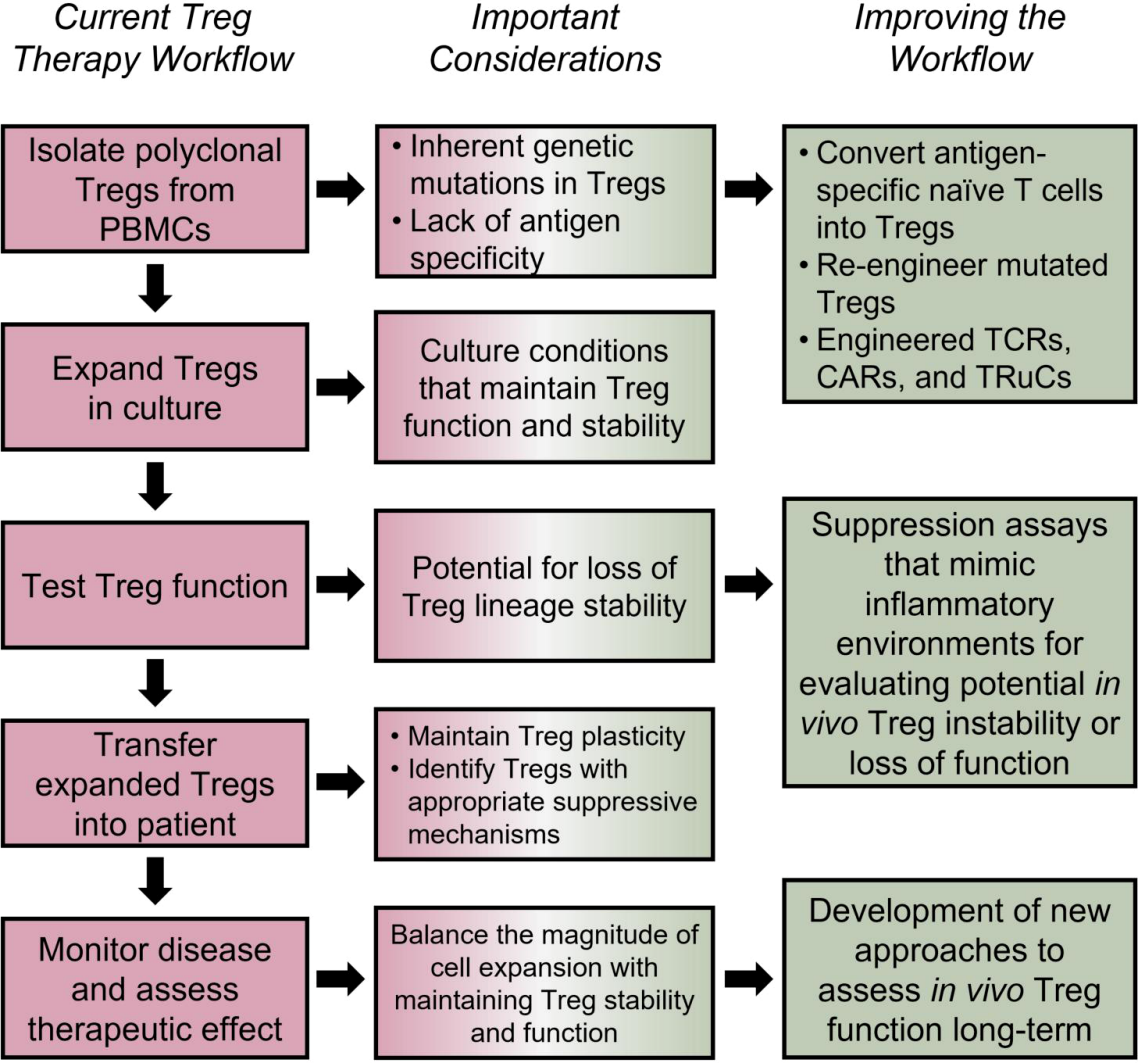
Tailoring Treg therapies for improved efficacy. Using human autologous Tregs is a promising approach for treatment of autoimmune and inflammatory disorders (196); however, the efficacy of such approaches depends on several factors. Loss of Treg suppressive capacity, stability, or stemness could be a side effect of *in vitro* expansion protocols (205–207). The potential inherent defects in Tregs, lack of antigen specificity (208–211), TSDR methylation status post expansion (206), and long-term functionality must also be considered. Potential solutions include engineering antigen specific TCRs (212), TRuCs (213–215), and CARs (216–219), utilizing Cas9/CRISPR technology for targeted demethylation of the TSDR (220), and using cytokine cocktails to optimize Treg expansion and functionality long-term (221, 222). Furthermore, clinical studies should be focused on accurately assessing the long-term *in vivo* Treg lineage stability, survival and disease-specific Treg suppressive mechanisms.

Another avenue to address Treg frequency is by isolating and expanding endogenous Tregs from T1D patients directly *in vitro* followed by adoptive transfer back into the patient (**Figure 4**) (196). One way to expand Tregs utilizes the IL-2 pathway. For example, several studies have used low-dose IL-2 as a way to expand Tregs *in vivo* and increase their suppressive function (223, 224). Careful dosing of IL-2 in this approach is critical since high-dose IL-2 also expands effector T cells and other immune cell populations. Recent studies have addressed this dosing issue and improved upon this approach by modifying the IL-2 cytokine so that it selectively binds to Tregs (225–227). Targeting the IL-2 pathway is logical, as the decrease in Tregs seen during NOD diabetes progression is thought to be due to dysregulated IL-2 production within the pancreatic islets leading to loss in Treg function and survival (158), and IL-2R dysfunction is implicated in development of T1D (9). However, combining IL-2 therapy with autologous polyclonal expanded Treg infusion can have the potential to induce more harm than good. When IL-2 and Tregs are concomitantly administered to T1D recipients, IL-2 induces the proliferation not only of Tregs, but also of potentially cytotoxic cells, highlighting the need for Treg specific IL-2 (228). Although, low-dose IL-2 was well tolerated and specifically expanded Tregs in individuals of other autoimmune diseases (229).

An additional caveat to Treg therapy is how Tregs may change during the manufacturing process, i.e., expansion of Tregs *ex vivo*. While *ex vivo*-expanded Tregs maintain suppressive capacity (205), they can also upregulate inflammatory effector T cell-associated cytokines, such as IFNγ, which can lead to loss of Treg stability (**Figure 4**) (207). Genome wide DNA methylation sequencing on Tregs undergoing *in vitro* expansion show increased methylation in enhancer and promoter regions of genes associated with T cell activation and function, as well as hypomethylation of genes associated with T cell exhaustion. These results are donor independent and are consistent throughout manufacturing runs (206), raising the question of whether Tregs expanded under current *in vitro* protocols are poised for long term function *in vivo*, regardless of their transcriptomic landscape or suppressive capacities at the end of expansion. Findings such as this could elucidate why current Treg therapies often fail to suppress disease long term. Fortunately, recent experiments have shown that the Cas9/CRISPR system can be used for targeted TET-mediated demethylation of the Treg TSDR (220); potentially providing a solution for Treg manufacturing complications. Indeed, simply using a chemical inducer of TSDR demethylation was shown to decrease NOD diabetes disease (230).

Another standard approach for *in vitro* expansion of T cells, including Tregs, is based on anti-CD3/CD28 crosslinking that leads to engagement of TCR and co-stimulatory pathways. However, strong and continuous TCR stimulation might result in loss of Treg stability or lead to Treg exhaustion. As an alternative to using anti-CD3/CD28, a combination of cytokines and CD28 superagonist antibodies (CD28SA) can induce robust Treg expansion while maintaining superior Treg stability (221, 222). Collectively, these findings suggest that a more tailored approach is necessary to create Treg-based treatments, and that increased Treg frequency, while helpful, needs to be accompanied by a high suppressive capacity in order to fully curtail disease.

## Antigen specificity in Treg therapy

Another important consideration for effective Treg therapy is their tissue antigen specificity, which was shown to be necessary for optimal Treg function in mouse models of T1D (209, 211). Indeed, islet auto-antigen specific, but not polyclonal Tregs transferred into NOD mice are capable of engrafting and expanding following anti-CD3 Ab treatment (208). This may be due to antigen specific Tregs’ ability to traffic to the site of autoimmune inflammation more efficiently than polyclonal Tregs. For example, a clinical trial that recently concluded in MS patients saw that *ex vivo* expanded polyclonal CD4+CD25^high^CD127-FoxP3+ Tregs injected intrathecally, but not intravenously, had the ability to reduce disease severity, suggesting that inflammatory signals alone are not sufficient for recruitment of Tregs to the autoimmune tissue (210). Various approaches have been in development to increase antigen specific Tregs. One approach involved expansion of antigen specific Tregs *in vitro* using CD8+ splenic dendritic cells presenting islet antigens. Islet-antigen specific Tregs generated using this method had the ability to suppress diabetogenic T cells (231). Antigen specific Tregs can also be induced directly *in vivo*, as was observed in a recent clinical trial that utilized the *in vivo* delivery of beta cell peptide antigens (232). One potential problem that exists with this approach, however, is that some patients have inherent defects in their Treg populations, and thus it may be difficult to increase the number of functional Tregs. To address a potential lack of Treg precursors, one approach is to insert an enhancer before the *FOXP3* coding region in bulk CD4 T cells (233). This approach overcomes epigenetic repression of the *FOXP3* gene and can be used on antigen-specific CD4 conventional T cells (**Figure 4**). In addition, these edited Tregs express Treg signature genes and have a similar suppressive potential as naturally derived tTregs (234).

Understanding and identifying various subpopulations of Tregs is an important step to improving Treg-based therapies for autoimmune diseases, as the ability to isolate highly functional Tregs would be beneficial in enriching potentially more efficacious Tregs. As an example, TIGIT+ human Tregs positively correlate with stable FOXP3 expression (demethylated TSDR) while CD226+ Tregs are associated with effector cytokine expression and increased TSDR methylation (235). Furthermore, additional Treg subpopulations have been identified, that may increase our understanding of Treg biology and function (236).

Another approach to conferring antigen specificity to Tregs is with engineered TCRs, TCR-fusion constructs (TRuCs), or chimeric antigen receptors (CARs) (**Figure 4**). As the name suggests, engineered TCR Tregs are Tregs transfected with an antigen-specific TCR, however this approach may not create TCRs with a high enough affinity to be effective in resolving autoimmunity (212). Alternatively, CD4+FOXP3+ T cells can be transduced with a high affinity CAR specific for an autoimmune antigen (216, 217). Current results suggest that CAR Tregs specific for autoimmune antigens can traffic to the correct tissue site and maintain suppressive function (218, 219). TRuCs on the other hand, are tissue-protein specific antibody fragments fused to TCR, allowing for antigen recognition to be combined with natural TCR signaling (214). This approach may be superior to CAR Tregs when there is low density of the antigen available at the tissue site (213, 215).

## Discussion

Understanding the genetic elements that lead to loss of regulatory T cell function in autoimmunity requires a foundational understanding of Treg function in a homeostatic environment. Control of Treg lineage and stability often revolve around the transcription factor FOXP3, although FOXP3 activity only accounts for a part of all Treg signature gene expression. Recent evidence has shown that FOXP3 expression and activity is tightly controlled through many different cis- and trans-regulatory factors including enhancer regions, transcription factor complexes, and epigenetic modifications. In turn, these regulatory factors can be influenced by the surrounding tissue environment, allowing for tight control of tolerance in healthy individuals. Thus, ultimate Treg function is a matter of both nature and nurture.

Genetic mutations leading to IPEX syndrome and polygenetic autoimmune susceptibilities revealed through GWAS analyses (9–11) converge on several pathways crucial to Treg stability and function and imply their dysregulation during autoimmunity. The dysregulation can be caused by mutations in *FOXP3* itself, mutations in Treg functional genes, or SNPs that affect regulatory elements such as enhancer regions or genes critical for proper Treg function. In addition, transcription factor complexes that associate with CNS regions of *FOXP3*, are another component that give Tregs a ‘manual’ for how they should function in maintaining immune tolerance. However, this so-called manual often becomes distorted or destroyed during pathological autoimmunity, which might be attributed to chronic inflammation present in the tissue environment.

We know that Tregs are poised to interact with their environment and to make functional changes in response to seemingly minute alterations; especially compared to their effector T cell counterparts. The ability for a lymphoid resident Treg to undergo transcriptional reprogramming in order to become a tissue Treg is only one example of such functional changes. Additional evidence can be found in the sensitivity Tregs have to IL-2 in their surrounding environment, and the ability of Tregs to utilize unique metabolites (237–239). The idea that Tregs are influenced by their environment is not novel; however, there is growing appreciation that the environment or so-called ‘nurture’ can impose permanent changes in Treg nature.

GWAS and other -omics studies point to Treg defects as a partial contribution to autoimmune susceptibility. However, the ultimate trigger that destabilizes the immune system and leads to autoimmunity is hard to define. Do Tregs become dysfunctional due to the tissue environment created during inflammation or autoimmune attack, or are they dysfunctional prior to the initial triggering event? Perhaps, Tregs in autoimmune patients may be poised for dysregulation, but are only partially impaired and progressively lose function in response to specific environmental changes. Perturbations in the environment might provoke a series of downstream events related to epigenetic and transcriptomic changes of Tregs; ultimately leading to a loss of function and self-tolerance.

## Author contributions

AR, DA, and MB conceptualized and wrote the manuscript. All authors contributed to the article and approved the submitted version.

## Acknowledgments

The work was supported by National Institutes of Health, R01 AI125301 to MB. Figures were generated using Biorender.com. The authors would like to thank Matt Bettini, Yi Jing, Viva Rase, and Nouf Aljobaily for their critical reading and insightful feedback during the preparation of this review.

## Conflict of interest

The authors declare that the research was conducted in the absence of any commercial or financial relationships that could be construed as a potential conflict of interest.

## Publisher’s note

All claims expressed in this article are solely those of the authors and do not necessarily represent those of their affiliated organizations, or those of the publisher, the editors and the reviewers. Any product that may be evaluated in this article, or claim that may be made by its manufacturer, is not guaranteed or endorsed by the publisher.
